# Supplementary material for: High-throughput generation and comparison of genome-scale metabolic models reveal strain-specific metabolic diversity in 439 Lactococcus strains
Source: mSystems. 2026 Mar 30;11(4):e01517-25. doi: 10.1128/msystems.01517-25 (PMC13098203; doi:10.1128/msystems.01517-25)
Supplement: Text S1 — Summary of updates to the L. cremoris MG1363 template model. [file msystems.01517-25-s0001.docx]

# Text S1 - Updates to the *L. cremoris* MG1363 template model

The published *L. cremoris* MG1363 model (Flahaut et al., 2013) was used as a template for generating the *L. lactis* and *cremoris* strain specific models in this study. Furthermore, the biomass reaction from this model, which was adapted from the biomass reaction developed by Oliveira et al., (2005) based on the biomass composition of *L. lactis* IL1403, was used as template biomass reaction for the models generated in this study. To utilize this model in combination with CarveMe (Machado et al., 2018) both as template and for the biomass reaction, the model needed to be compatible with the universal bacterial model, which is the database for model elements utilized by CarveMe.

## Updating the reactions and metabolites to match the universal bacterial model

Since this model was already included in the original universal bacterial model as published by Machado et al., (2018), most reactions and metabolites were already represented in the universal bacterial model. However, since the universal bacterial model was updated in this study, the *L. cremoris* template model needed to be updated to ensure metabolites and reactions matched with the equivalents used in the universal bacterial model.

Metabolites were matched with their equivalents in the universal bacterial model if available. If the metabolite ID, formula, or charge differed from the ID, formula or charge used in the universal bacterial model, the metabolite was updated to match with the universal bacterial model. Reactions were also matched with their equivalents in the universal bacterial model if available. If the reaction ID in the model differed from the universal bacterial model, the ID was updated to match the universal bacterial model. A detailed overview of all changes to metabolites and reactions can be found in tables S4 and S5.

## Updating the biomass reaction

### Replacing organism specific biomass intermediate components

The *L. cremoris* MG1363 model also contained a *Lactoccocus* species specific biomass reaction, which was adapted from Oliveira et al., (2005). This biomass reaction utilizes organism specific intermediate components for different cell constituents: proteins, DNA, RNA, polysaccharides, and lipids. The use of organism specific intermediates is an insightful way to represent the experimentally determined composition of biomass from these constituents and conversely the composition of each of these constituents. The formation of each of these constituents is represented by separate organism specific reactions, which have been developed to reflect the experimentally determined composition in the stoichiometry of the different metabolites that are combined into this organism specific biomass constituent (Figure A1a). However, since this approach requires several additional organism specific reactions for the formation of each of the biomass constituents in addition to the final biomass reaction, this approach is not compatible with CarveMe and the universal bacterial model, as the latter utilizes a single biomass reaction which contains only universal metabolites, representing their contribution to the total composition of the biomass of the organism of interest. To take advantage of the biomass reaction from the MG1363 model, representing the biomass composition of *Lactococcus*, the reaction was updated to match with the single biomass reaction format utilized in the universal model. To do so each *Lactococcus* specific biomass constituent in the biomass reaction was replaced with the universal metabolites used to form this biomass constituent.

The biomass reaction from the MG1363 model is shown below in equation 1:

[1] $0.0064 CPS_{LLA}+ 0.00074 DNA_{LLA}+0.00015 LTAAlaGal_{LLA}+0.119 PG+0.004201 PROT_{LLA}+0.00329 RNA_{LLA}+ 0.000138 CLPN_{LLA}+9.6e^{-05} d12dg_{LLA}+ 1.3e^{-05} \mathrm{lyspg}_{\mathrm{LLA}} + 1.3e^{-05} m12\mathrm{dg}_{\mathrm{LLA}} +6.1e-05 \mathrm{pg}_{\mathrm{LLA}}+ 1e-05 \mathrm{thf}+ 1e-05 \mathrm{thmpp}+ 0.0002 \mathrm{udcpdp}+0.0002 CoA+ 0.002 nad+39.4 ATP+39.4 H_{2}O\to39.4 adp + 39.4 h^{+} + 39.4 pi$

For each of the *Lactococcus* specific biomass constituents in the biomass reaction, the reaction to form this *Lactococcus* specific component was used as a basis to replace the component with the universal metabolites it consists off (see figure A1 for illustration). The tables A1-10 specify the universal metabolites that each the *Lactococcus* specific biomass intermediate components were replaced by.


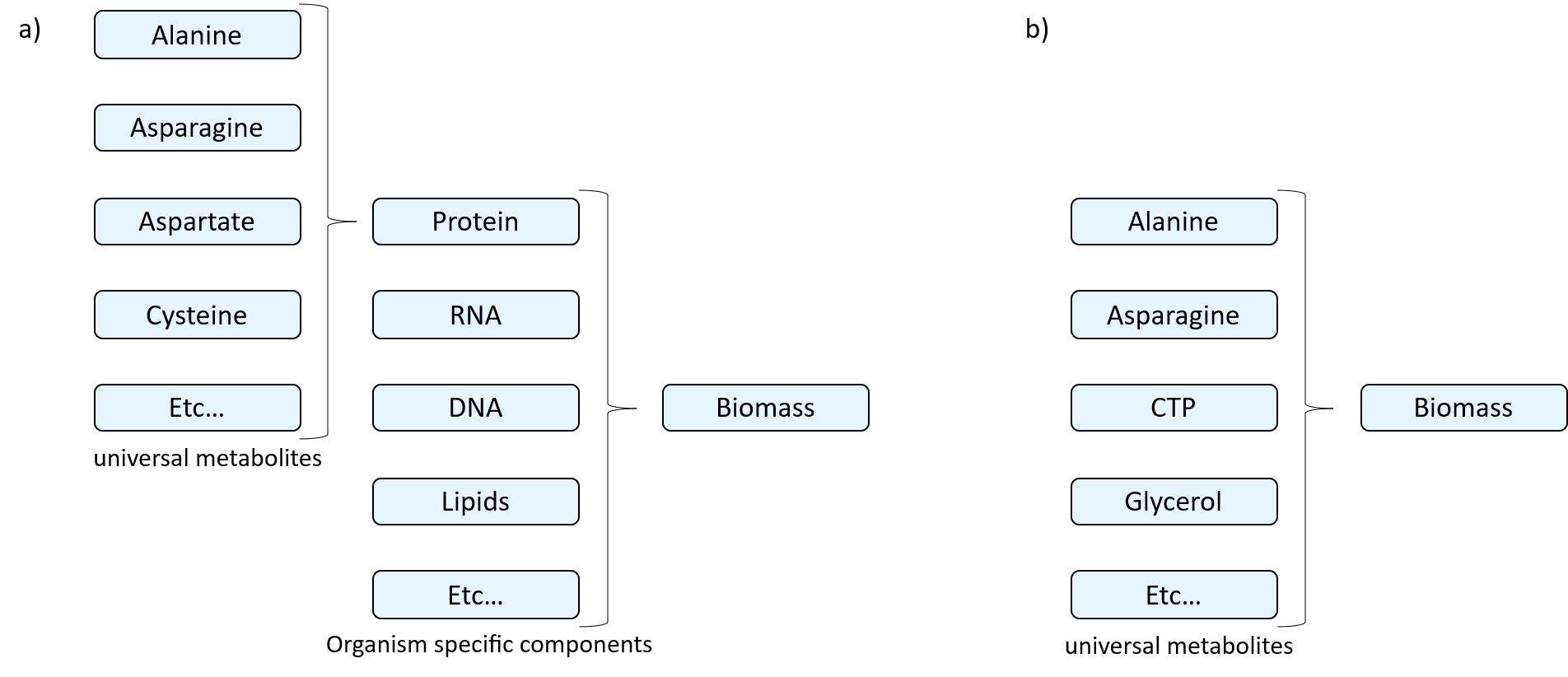


Figure A1 a) Simplified overview of the formulation of the biomass reaction modeling approach used in the published L. cremoris MG1363 model (Flahaut et al., 2013) b) simplified overview of the formulation of a single biomass reaction containing only universal metabolites.

Table A1: Breakdown of the CPS –Polysacccharide units into the universal metabolites that constitute the L. cremoris specific biomass component.

| ID | Name | Coeff. original biomass reaction | Coeff. updated biomass reaction | Coeff. in synthesis reaction | Formula | Charge |
| --- | --- | --- | --- | --- | --- | --- |
| **CPS_LLA** | **Polysaccharide units Lactis specific** | **-6,40E-03** | **0** | **1,00** | **C_24_H_47_O_26_P_1_** | **-2** |
| dtdp6dm | DTDP 6 deoxy L mannose | 0 | -1,53E-01 | -1,00 | C_16_H_24_N_2_O_15_P_2_ | -2 |
| h2o | H2O | -3,94E+01 | -5,00E+01 | -4,00 | H_2_O | 0 |
| udpg | UDPglucose | 0 | -3,57E-01 | -2,00 | C_15_H_22_N_2_O_17_P_2_ | -2 |
| udpgal | UDPgalactose | 0 | -3,00E-01 | -1,00 | C_15_H_22_N_2_O_17_P_2_ | -2 |
| dtdp | DTDP | 0 | 1,53E-01 | 1,00 | C_10_H_13_N_2_O_11_P_2_ | -3 |
| h | H+ | 3,94E+01 | 5,01E+01 | 5,00 | H | 1 |
| udp | UDP | 0 | 6,58E-01 | 2,00 | C_9_H_11_N_2_O_12_P_2_ | -3 |
| ump | UMP | 0 | 0 | 1,00 | C_9_H_11_N_2_O_9_P | -2 |

Table A2: Breakdown of the DNA component into the universal metabolites that constitute the L. cremoris specific biomass component.

| ID | Name | Coeff. original biomass reaction | Coeff. updated biomass reaction | Coeff. in synthesis reaction | Formula | Charge |
| --- | --- | --- | --- | --- | --- | --- |
| **DNA_LLA** | **DNA composition lactis specific** | **-7,40E-04** | **0** | **1,00E-02** | **C_982_H_1132_N_368_O_600_P_100_** | **-100** |
| atp | ATP | -3,94E+01 | -4,60E+01 | -1,37 | C_10_H_12_N_5_O_13_P_3_ | -4 |
| datp | DATP | 0 | -2,37E-02 | -3,20E-01 | C_10_H_12_N_5_O_12_P_3_ | -4 |
| dctp | DCTP | 0 | -1,33E-02 | -1,80E-01 | C_9_H_12_N_3_O_13_P_3_ | -4 |
| dgtp | DGTP | 0 | -1,33E-02 | -1,80E-01 | C_10_H_12_N_5_O_13_P_3_ | -4 |
| dttp | DTTP | 0 | -2,37E-02 | -3,20E-01 | C_10_H_13_N_2_O_14_P_3_ | -4 |
| h2o | H2O | -3,94E+01 | -5,00E+01 | -1,37 | H_2_O | 0 |
| adp | ADP | 3,94E+01 | 4,59E+01 | 1,37 | C_10_H_12_N_5_O_10_P_2_ | -3 |
| h | H+ | 3,94E+01 | 5,01E+01 | 1,37 | H | 1 |
| pi | Phosphate | 3,94E+01 | 4,95E+01 | 1,37 | HO_4_P | -2 |
| ppi | Diphosphate | 0 | 9,54 | 1,00 | HO_7_P_2_ | -3 |

Table A3: Breakdown of the RNA component into the universal metabolites that constitute the L. cremoris specific biomass component.

| ID | Name | Coeff. original biomass reaction | Coeff. updated biomass reaction | Coeff. in synthesis reaction | Formula | Charge |
| --- | --- | --- | --- | --- | --- | --- |
| **RNA_LLA** | **RNA biosynthesis lactis** | **-3,29E-03** | **0** | **1,00E-02** | **C_958_H_1078_N_394_O_696_P_100_** | **-100** |
| atp | ATP | -3,94E+01 | -4,60E+01 | -6,60E-01 | C_10_H_12_N_5_O_13_P_3_ | -4 |
| ctp | CTP | 0 | -3,41E-01 | -2,00E-01 | C_9_H_12_N_3_O_14_P_3_ | -4 |
| gtp | GTP | 0 | -8,51 | -3,20E-01 | C_10_H_12_N_5_O_14_P_3_ | -4 |
| h2o | H2O | -3,94E+01 | -5,00E+01 | -4,00E-01 | H_2_O | 0 |
| utp | UTP | 0 | -7,24E-02 | -2,20E-01 | C_9_H_11_N_2_O_15_P_3_ | -4 |
| adp | ADP | 3,94E+01 | 4,59E+01 | 4,00E-01 | C_10_H_12_N_5_O_10_P_2_ | -3 |
| h | H+ | 3,94E+01 | 5,01E+01 | 4,00E-01 | H | 1 |
| pi | Phosphate | 3,94E+01 | 4,95E+01 | 4,00E-01 | HO_4_P | -2 |
| ppi | Diphosphate | 0 | 9,54 | 1,00 | HO_7_P_2_ | -3 |

Table A4: Breakdown of the protein component into the universal metabolites that constitute the L. cremoris specific biomass component.

| ID | Name | Coeff. original biomass reaction | Coeff. updated biomass reaction | Coeff. in synthesis reaction | Formula | Charge |
| --- | --- | --- | --- | --- | --- | --- |
| **PROT_LLA_v3** | **Protein for biomass lactis specific v3** | **-4,20E-03** | **0** | **1,00E-03** | **C_4869_H_7804_N_1364_O_1390_S_59_** | **46** |
| atp | ATP | -3,94E+01 | -4,60E+01 | -1,36 | C_10_H_12_N_5_O_13_P_3_ | -4 |
| gtp | GTP | 0 | -8,51 | -2,00 | C_10_H_12_N_5_O_14_P_3_ | -4 |
| h2o | H2O | -3,94E+01 | -5,00E+01 | -2,31 | H_2_O | 0 |
| adp | ADP | 3,94E+01 | 4,59E+01 | 3,06E-01 | C_10_H_12_N_5_O_10_P_2_ | -3 |
| gdp | GDP | 0 | 8,40 | 2,00 | C_10_H_12_N_5_O_11_P_2_ | -3 |
| h | H+ | 3,94E+01 | 5,01E+01 | 3,31 | H | 1 |
| pi | Phosphate | 3,94E+01 | 4,95E+01 | 2,31 | HO_4_P | -2 |
| ala__L | L-Alanine | 0 | -3,65E-01 | -8,70E-02 | C_3_H_7_NO_2_ | 0 |
| amp | AMP | 0 | 0 | 1,06 | C_10_H_12_N_5_O_7_P | -2 |
| ppi | Diphosphate | 0 | 9,54 | 1,06 | HO_7_P_2_ | -3 |
| arg__L | L-Arginine | 0 | -1,76E-01 | -4,20E-02 | C_6_H_15_N_4_O_2_ | 1 |
| asn__L | L-Asparagine | 0 | -1,97E-01 | -4,70E-02 | C_4_H_8_N_2_O_3_ | 0 |
| asp__L | L-Aspartate | 0 | -1,97E-01 | -4,70E-02 | C_4_H_6_NO_4_ | -1 |
| cys__L | L-Cysteine | 0 | -2,06E-01 | -4,90E-02 | C_3_H_7_NO_2_S | 0 |
| nh4 | Ammonium | 0 | -2,31E-01 | -5,50E-02 | H_4_N | 1 |
| glu__L | L-Glutamate | 0 | -2,31E-01 | -5,50E-02 | C_5_H_8_NO_4_ | -1 |
| gly | Glycine | 0 | -2,48E-01 | -5,90E-02 | C_2_H_5_NO_2_ | 0 |
| his__L | L-Histidine | 0 | -7,56E-02 | -1,80E-02 | C_6_H_9_N_3_O_2_ | 0 |
| ile__L | L-Isoleucine | 0 | -2,44E-01 | -5,80E-02 | C_6_H_13_NO_2_ | 0 |
| leu__L | L-Leucine | 0 | -3,70E-01 | -8,80E-02 | C_6_H_13_NO_2_ | 0 |
| lys__L | L-Lysine | 0 | -2,37E-01 | -5,60E-02 | C_6_H_15_N_2_O_2_ | 1 |
| met__L | L-Methionine | 0 | -8,40E-02 | -2,00E-02 | C_5_H_11_NO_2_S | 0 |
| phe__L | L-Phenylalanine | 0 | -1,68E-01 | -4,00E-02 | C_9_H_11_NO_2_ | 0 |
| pro__L | L-Proline | 0 | -1,81E-01 | -4,30E-02 | C_5_H_9_NO_2_ | 0 |
| ser__L | L-Serine | 0 | -2,48E-01 | -5,90E-02 | C_3_H_7_NO_3_ | 0 |
| thr__L | L-Threonine | 0 | -2,77E-01 | -6,60E-02 | C_4_H_9_NO_3_ | 0 |
| trp__L | L-Tryptophan | 0 | -3,36E-02 | -8,00E-03 | C_11_H_12_N_2_O_2_ | 0 |
| tyr__L | L-Tyrosine | 0 | -1,26E-01 | -3,00E-02 | C_9_H_11_NO_3_ | 0 |
| val__L | L-Valine | 0 | -3,07E-01 | -7,30E-02 | C_5_H_11_NO_2_ | 0 |
| gln__L | L-Glutamine | 0 | -2,31E-01 | -5,50E-02 | C_5_H_10_N_2_O_3_ | 0 |

Table A5: Breakdown of the Lipoteichoic acid component into the universal metabolites that constitute the L. cremoris specific biomass component.

| ID | Name | Coeff. original biomass reaction | Coeff. updated biomass reaction | Coeff. in synthesis reaction | Formula | Charge |
| --- | --- | --- | --- | --- | --- | --- |
| **LTAAlaGal_LLA** | **Lipoteichoic acid n16 with 0 38 ala and 062 gal** | **-1,50E-04** | **0** | **1,00E-02** | **C_17400_H_31998_N_600_O_15000_P_1600_** | **0** |
| udpgal | UDPgalactose | 0 | -3,00E-01 | -9,80 | C_15_H_22_N_2_O_17_P_2_ | -2 |
| h | H+ | 3,94E+01 | 5,01E+01 | 1,18E+01 | H | 1 |
| udp | UDP | 0 | 6,58E-01 | 1,18E+01 | C_9_H_11_N_2_O_12_P_2_ | -3 |
| ala__D | D-Alanine | 0 | -9,00E-02 | -6,00 | C_3_H_7_NO_2_ | 0 |
| atp | ATP | -3,94E+01 | -4,60E+01 | -6,00 | C_10_H_12_N_5_O_13_P_3_ | -4 |
| adp | ADP | 3,94E+01 | 4,59E+01 | 6,00 | C_10_H_12_N_5_O_10_P_2_ | -3 |
| pi | Phosphate | 3,94E+01 | 4,95E+01 | 7,00 | HO_4_P | -2 |
| udpg | UDPglucose | 0 | -3,57E-01 | -2,00 | C_15_H_22_N_2_O_17_P_2_ | -2 |
| glyc3p | Glycerol 3-phosphate | 0 | -3,22E-01 | -1,70E+01 | C_3_H_7_O_6_P | -2 |
| h2o | H2O | -3,94E+01 | -5,00E+01 | -1,00 | H_2_O | 0 |
| 2chdeacp | Cis Hexadec 2 enoyl acyl carrier protein | 0 | -3,65E-03 | -6,00E-02 | C_400_H_631_N_96_O_143_P_1_S_3_ | -1 |
| 2cocdacp | Cis Octadec 2 enoyl acyl carrier protein | 0 | -5,36E-02 | -8,80E-01 | C_402_H_635_N_96_O_143_P_1_S_3_ | -1 |
| 2ctdeacp | Cis Tetradec 2 enoyl acyl carrier protein | 0 | -6,09E-04 | -1,00E-02 | C_398_H_627_N_96_O_143_PS_3_ | -1 |
| cpocdacp | Cyclopropanoyl octadecanoyl acyl carrier protein | 0 | -1,58E-02 | -2,60E-01 | C_403_H_637_N_96_O_143_P_1_S_3_ | -1 |
| hdeACP | Hexadecanoyl acyl carrier protein | 0 | -3,59E-02 | -5,90E-01 | C_400_H_633_O_143_N_96_P_1_S_3_ | -1 |
| ocdacp | Octadecanoyl acyl carrier protein | 0 | -1,22E-03 | -2,00E-02 | C_402_H_637_N_96_O_143_P_1_S_3_ | -1 |
| tdeACP | Tetradecanoyl acyl carrier protein | 0 | -1,10E-02 | -1,80E-01 | C_398_H_629_O_143_N_96_P_1_S_3_ | -1 |
| ACP | Acyl carrier protein | 0 | 1,22E-01 | 2,00 | C_384_H_603_N_96_O_142_P_1_S_3_ | -1 |
| ctp | CTP | 0 | -3,41E-01 | -1,60E+01 | C_9_H_12_N_3_O_14_P_3_ | -4 |
| ppi | Diphosphate | 0 | 9,54 | 1,60E+01 | HO_7_P_2_ | -3 |
| cmp | CMP | 0 | 0 | 1,60E+01 | C_9_H_12_N_3_O_8_P | -2 |

Table A6: Breakdown of the Cardiolipin acid component into the universal metabolites that constitute the L. cremoris specific biomass component.

| ID | Name | Coeff. original biomass reaction | Coeff. updated biomass reaction | Coeff. in synthesis reaction | Formula | Charge |
| --- | --- | --- | --- | --- | --- | --- |
| **clpn_LLA** | **Cardiolipin lactis specific** | **-1,38E-04** | **0** | **1,00E-02** | **C_7740_H_14396_O_1700_P_200_** | -200 |
| glyc | Glycerol | 0 | 0 | 1,00 | C_3_H_8_O_3_ | 0 |
| h2o | H2O | -3,94E+01 | -5,00E+01 | -2,00 | H_2_O | 0 |
| pi | Phosphate | 3,94E+01 | 4,95E+01 | 2,00 | HO_4_P | -2 |
| glyc3p | Glycerol 3-phosphate | 0 | -3,22E-01 | -4,00 | C_3_H_7_O_6_P | -2 |
| cmp | CMP | 0 | 0 | 2,00 | C_9_H_12_N_3_O_8_P | -2 |
| ctp | CTP | 0 | -3,41E-01 | -2,00 | C_9_H_12_N_3_O_14_P_3_ | -4 |
| ppi | Diphosphate | 0 | 9,54 | 2,00 | HO_7_P_2_ | -3 |
| 2chdeacp | Cis Hexadec 2 enoyl acyl carrier protein | 0 | -3,65E-03 | -1,20E-01 | C_400_H_631_N_96_O_143_P_1_S_3_ | -1 |
| 2cocdacp | Cis Octadec 2 enoyl acyl carrier protein | 0 | -5,36E-02 | -1,76 | C_402_H_635_N_96_O_143_P_1_S_3_ | -1 |
| 2ctdeacp | Cis Tetradec 2 enoyl acyl carrier protein | 0 | -6,09E-04 | -2,00E-02 | C_398_H_627_N_96_O_143_P_1_S_3_ | -1 |
| cpocdacp | Cyclopropanoyl octadecanoyl acyl carrier protein | 0 | -1,58E-02 | -5,20E-01 | C_403_H_637_N_96_O_143_P_1_S_3_ | -1 |
| hdeACP | Hexadecanoyl acyl carrier protein | 0 | -3,59E-02 | -1,18 | C_400_H_633_O_143_N_96_P_1_S_3_ | -1 |
| ocdacp | Octadecanoyl acyl carrier protein | 0 | -1,22E-03 | -4,00E-02 | C_402_H_637_N_96_O_143_P_1_S_3_ | -1 |
| tdeACP | Tetradecanoyl acyl carrier protein | 0 | -1,10E-02 | -3,60E-01 | C_398_H_629_O_143_N_96_P_1_S_3_ | -1 |
| ACP | Acyl carrier protein | 0 | 1,22E-01 | 4,00 | C_384_H_603_N_96_O_142_P_1_S3 | -1 |

Table A7: Breakdown of the Diglucosyl 1-2-diacylglycerol acid component into the universal metabolites that constitute the L. cremoris specific biomass component.

| ID | Name | Coeff. original biomass reaction | Coeff. updated biomass reaction | Coeff. in synthesis reaction | Formula | Charge |
| --- | --- | --- | --- | --- | --- | --- |
| **d12dg_LLA** | **Diglucosyl 1 2 diacylglycerol** | **-9,60E-05** | **0** | **1,00E-02** | **C_4920_H_8998_O_1500_** | **0** |
| udpg | UDPglucose | 0 | -3,57E-01 | -2,00 | C_15_H_22_N_2_O_17_P_2_ | -2 |
| h | H+ | 3,94E+01 | 5,01E+01 | 2,00 | H | 1 |
| udp | UDP | 0 | 6,58E-01 | 2,00 | C_9_H_11_N_2_O_12_P_2_ | -3 |
| glyc3p | Glycerol 3-phosphate | 0 | -3,22E-01 | -1,00 | C_3_H_7_O_6_P | -2 |
| h2o | H2O | -3,94E+01 | -5,00E+01 | -1,00 | H_2_O | 0 |
| pi | Phosphate | 3,94E+01 | 4,95E+01 | 1,00 | HO_4_P | -2 |
| 2chdeacp | Cis Hexadec 2 enoyl acyl carrier protein | 0 | -3,65E-03 | -6,00E-02 | C_400_H_631_N_96_O_143_P_1_S_3_ | -1 |
| 2cocdacp | Cis Octadec 2 enoyl acyl carrier protein | 0 | -5,36E-02 | -8,80E-01 | C_402_H_635_N_96_O_143_P_1_S_3_ | -1 |
| 2ctdeacp | Cis Tetradec 2 enoyl acyl carrier protein | 0 | -6,09E-04 | -1,00E-02 | C_398_H_627_N_96_O_143_P_1_S_3_ | -1 |
| cpocdacp | Cyclopropanoyl octadecanoyl acyl carrier protein | 0 | -1,58E-02 | -2,60E-01 | C_403_H_637_N_96_O_143_P_1_S_3_ | -1 |
| hdeACP | Hexadecanoyl acyl carrier protein | 0 | -3,59E-02 | -5,90E-01 | C_400_H_633_O_143_N_96_P_1_S_3_ | -1 |
| ocdacp | Octadecanoyl acyl carrier protein | 0 | -1,22E-03 | -2,00E-02 | C_402_H_637_N_96_O_143_P_1_S_3_ | -1 |
| tdeACP | Tetradecanoyl acyl carrier protein | 0 | -1,10E-02 | -1,80E-01 | C_398_H_629_O_143_N_96_P_1_S_3_ | -1 |
| ACP | Acyl carrier protein | 0 | 1,22E-01 | 2,00 | C_384_H_603_N_96_O_142_P_1_S_3_ | -1 |

Table A8: Breakdown of the 1-lysyl phosphatidyl glycerol component into the universal metabolites that constitute the L. cremoris specific biomass component

| ID | Name | Coeff. original biomass reaction | Coeff. updated biomass reaction | Coeff. in synthesis reaction | Formula | Charge |
| --- | --- | --- | --- | --- | --- | --- |
| **lyspg_LLA** | **1 lysyl phosphatidyl glycerol lactis specific** | **-1,30E-05** | **0** | **1,00E-02** | **C_4620_H_8898_N_200_O_1100_P_100_** | **0** |
| h2o | H2O H2O | -3,94E+01 | -5,00E+01 | -1,00 | H_2_O | 0 |
| pi | Phosphate | 3,94E+01 | 4,95E+01 | 1,00 | HO_4_P | -2 |
| glyc3p | Glycerol 3-phosphate | 0 | -3,22E-01 | -2,00 | C_3_H_7_O_6_P | -2 |
| cmp | CMP | 0 | 0 | 1,00 | C_9_H_12_N_3_O_8_P | -2 |
| ctp | CTP | 0 | -3,41E-01 | -1,00 | C_9_H_12_N_3_O_14_P_3_ | -4 |
| ppi | Diphosphate | 0 | 9,54 | 2,00 | HO_7_P_2_ | -3 |
| 2chdeacp | Cis Hexadec 2 enoyl acyl carrier protein | 0 | -3,65E-03 | -6,00E-02 | C_400_H_631_N_96_O_143_P_1_S_3_ | -1 |
| 2cocdacp | Cis Octadec 2 enoyl acyl carrier protein | 0 | -5,36E-02 | -8,80E-01 | C_402_H_635_N_96_O_143_P_1_S_3_ | -1 |
| 2ctdeacp | Cis Tetradec 2 enoyl acyl carrier protein | 0 | -6,09E-04 | -1,00E-02 | C_398_H_627_N_96_O_143_P_1_S_3_ | -1 |
| cpocdacp | Cyclopropanoyl octadecanoyl acyl carrier protein | 0 | -1,58E-02 | -2,60E-01 | C_403_H_637_N_96_O_143_P_1_S_3_ | -1 |
| hdeACP | Hexadecanoyl acyl carrier protein | 0 | -3,59E-02 | -5,90E-01 | C_400_H_633_O_143_N_96_P_1_S_3_ | -1 |
| ocdacp | Octadecanoyl acyl carrier protein | 0 | -1,22E-03 | -2,00E-02 | C_402_H_637_N_96_O_143_P_1_S_3_ | -1 |
| tdeACP | Tetradecanoyl acyl carrier protein | 0 | -1,10E-02 | -1,80E-01 | C_398_H_629_O_143_N_96_P_1_S_3_ | -1 |
| ACP | Acyl carrier protein | 0 | 1,22E-01 | 2,00 | C_384_H_603_N_96_O_142_P_1_S_3_ | -1 |
| atp | ATP | -3,94E+01 | -4,60E+01 | -1,00 | C_10_H_12_N_5_O_13_P_3_ | -4 |
| lys__L | L-Lysine | 0 | -2,37E-01 | -1,00 | C_6_H_15_N_2_O_2_ | 1 |
| amp | AMP | 0 | 0 | 1,00 | C_10_H_12_N_5_O_7_P | -2 |
| h | H+ | 3,94E+01 | 5,01E+01 | 1,00 | H | 1 |

Table A9: Breakdown of the Monoglycosyl 1-2-diacylglycerol component into the universal metabolites that constitute the L. cremoris specific biomass component

| ID | Name | Coeff. original biomass reaction | Coeff. updated biomass reaction | Coeff. in synthesis reaction | Formula | Charge |
| --- | --- | --- | --- | --- | --- | --- |
| **m12dg_LLA** | **Monoglucosyl 1 2 diacylglycerol** | **-1,30E-05** | **0** | **1,00E-02** | **C_4320_H_7998_O_1000_** | **0** |
| udpg | UDPglucose | 0 | -3,57E-01 | -1,00 | C_15_H_22_N_2_O_17_P_2_ | -2 |
| h | H+ | 3,94E+01 | 5,01E+01 | 1,00 | H | 1 |
| udp | UDP | 0 | 6,58E-01 | 1,00 | C_9_H_11_N_2_O_12_P_2_ | -3 |
| glyc3p | Glycerol 3-phosphate | 0 | -3,22E-01 | -1,00 | C_3_H_7_O_6_P | -2 |
| h2o | H2O H2O | -3,94E+01 | -5,00E+01 | -1,00 | H_2_O | 0 |
| pi | Phosphate | 3,94E+01 | 4,95E+01 | 1,00 | HO_4_P | -2 |
| 2chdeacp | Cis Hexadec 2 enoyl acyl carrier protein | 0 | -3,65E-03 | -6,00E-02 | C_400_H_631_N_96_O_143_P_1_S_3_ | -1 |
| 2cocdacp | Cis Octadec 2 enoyl acyl carrier protein | 0 | -5,36E-02 | -8,80E-01 | C_402_H_635_N_96_O_143_P_1_S_3_ | -1 |
| 2ctdeacp | Cis Tetradec 2 enoyl acyl carrier protein | 0 | -6,09E-04 | -1,00E-02 | C_398_H_627_N_96_O_143_P_1_S_3_ | -1 |
| cpocdacp | Cyclopropanoyl octadecanoyl acyl carrier protein | 0 | -1,58E-02 | -2,60E-01 | C_403_H_637_N_96_O_143_P_1_S_3_ | -1 |
| hdeACP | Hexadecanoyl acyl carrier protein | 0 | -3,59E-02 | -5,90E-01 | C_400_H_633_O_143_N_96_P_1_S_3_ | -1 |
| ocdacp | Octadecanoyl acyl carrier protein | 0 | -1,22E-03 | -2,00E-02 | C_402_H_637_N_96_O_143_P_1_S_3_ | -1 |
| tdeACP | Tetradecanoyl acyl carrier protein | 0 | -1,10E-02 | -1,80E-01 | C_398_H_629_O_143_N_96_P_1_S_3_ | -1 |
| ACP | Acyl carrier protein | 0 | 1,22E-01 | 2,00 | C_384_H_603_N_96_O_142_P_1_S_3_ | -1 |

Table A10: Breakdown of the Phospatidylglycerol component into the universal metabolites that constitute the L. cremoris specific biomass component

| ID | Name | Coeff. original biomass reaction | Coeff. updated biomass reaction | Coeff. in synthesis reaction | Formula | Charge |
| --- | --- | --- | --- | --- | --- | --- |
| **pg_LLA** | **Phospatidylglycerol Lactis specific** | **-6,10E-05** | **0** | **1,00E-02** | **C_4020_H_7598_O_1000_P_100_** | **-100** |
| h2o | H2O | -3,94E+01 | -5,00E+01 | -1,00 | H_2_O | 0 |
| pi | Phosphate | 3,94E+01 | 4,95E+01 | 1,00 | HO_4_P | -2 |
| glyc3p | Glycerol 3-phosphate | 0 | -3,22E-01 | -2,00 | C_3_H_7_O_6_P | -2 |
| cmp | CMP | 0 | 0 | 1,00 | C_9_H_12_N_3_O_8_P | -2 |
| ctp | CTP | 0 | -3,41E-01 | -1,00 | C_9_H_12_N_3_O_14_P_3_ | -4 |
| ppi | Diphosphate | 0 | 9,54 | 1,00 | HO_7_P_2_ | -3 |
| 2chdeacp | Cis Hexadec 2 enoyl acyl carrier protein | 0 | -3,65E-03 | -6,00E-02 | C_400_H_631_N_96_O_143_P_1_S_3_ | -1 |
| 2cocdacp | Cis Octadec 2 enoyl acyl carrier protein | 0 | -5,36E-02 | -8,80E-01 | C_402_H_635_N_96_O_143_P_1_S_3_ | -1 |
| 2ctdeacp | Cis Tetradec 2 enoyl acyl carrier protein | 0 | -6,09E-04 | -1,00E-02 | C_398_H_627_N_96_O_143_P_1_S_3_ | -1 |
| cpocdacp | Cyclopropanoyl octadecanoyl acyl carrier protein | 0 | -1,58E-02 | -2,60E-01 | C_403_H_637_N_96_O_143_P_1_S_3_ | -1 |
| hdeACP | Hexadecanoyl acyl carrier protein | 0 | -3,59E-02 | -5,90E-01 | C_400_H_633_O_143_N_96_P_1_S_3_ | -1 |
| ocdacp | Octadecanoyl acyl carrier protein | 0 | -1,22E-03 | -2,00E-02 | C_402_H_637_N_96_O_143_P_1_S_3_ | -1 |
| tdeACP | Tetradecanoyl acyl carrier protein | 0 | -1,10E-02 | -1,80E-01 | C_398_H_629_O_143_N_96_P_1_S_3_ | -1 |
| ACP | Acyl carrier protein | 0 | 1,22E-01 | 2,00 | C_384_H_603_N_96_O_142_P_1_S_3_ | -1 |

### Adding minerals and vitamins to the biomass reaction

Lastly, the utilization of several minerals and vitamins that are known to be essential for *L.* *lactis* and *cremoris* in growth media was not included in the MG1363 model or biomass reaction. These metabolites are typically only required in trace amounts and only make up a very small amount of the total biomass composition. To that end, it can be hard to quantify their presence in the biomass composition. Since these minerals and vitamins are known to be essential medium components for *L. lactis* and *cremoris* strains, including them in the biomass reaction can be important when utilizing the models to explore different media compositions and screen for essentiality, especially for vitamins that can be synthesized by some strains, but are essential medium components for others. Furthermore, adding vitamins to the biomass composition allows to consider vitamins as potential metabolites for crossfeeding between strains. Since quantification of these metabolites is difficult, they have been added using the same coefficient as in the general biomass reaction used in the universal bacterial model from CarveMe (Machado et al., 2018). See table A11 for an overview of vitamins and minerals included in the biomass reaction.

Table A11: Overview of the vitamins and minerals added to the biomass reaction based on experimental proof of their essentiality in medium for growth.

| Metabolite ID | Name | Formula | Charge | Biomass coef. (mmol/g) | Reference |
| --- | --- | --- | --- | --- | --- |
| ca2 | Calcium | Ca | 2 | 5,21E-03 | (Goel et al., 2012) |
| k | Potassium | K | 1 | 1,95E-01 | (Goel et al., 2012) |
| mg2 | Magnesium | Mg | 2 | 8,68E-03 | (Goel et al., 2012) |
| mn2 | Manganese | Mn | 2 | 6,91E-04 | (Goel et al., 2012) |
| so4 | Sulfate | O_4_S | -2 | 4,34E-03 | (Goel et al., 2012) |
| pydx5p | Pyridoxal 5'-phosphate | C_8_H_8_NO_6_P | -2 | 2,23E-04 | (Cocaign-Bousquet et al., 1995) |
| ribflv | Riboflavin | C_17_H_19_N_4_O_6_ | -1 | 2,23E-04 | (Cocaign-Bousquet et al., 1995) |
| zn2 | Zinc | Zn | 2 | 3,41E-04 | (Goel et al., 2012) |

## References:

Cocaign-Bousquet, M., Garrigues, C., Novak, L., Lindley, N. D., & Loublere, P. (1995). Rational development of a simple synthetic medium for the sustained growth of *Lactococcus lactis*. *Journal of Applied Bacteriology*, *79*(1), 108–116. https://doi.org/10.1111/j.1365-2672.1995.tb03131.x

Flahaut, N. A. L., Wiersma, A., Van De Bunt, B., Martens, D. E., Schaap, P. J., Sijtsma, L., Dos Santos, V. A. M., & De Vos, W. M. (2013). Genome-scale metabolic model for Lactococcus lactis MG1363 and its application to the analysis of flavor formation. *Applied Microbiology and Biotechnology*, *97*(19), 8729–8739. https://doi.org/10.1007/s00253-013-5140-2

Goel, A., Santos, F., de Vos, W. M., Teusink, B., & Molenaar, D. (2012). Standardized assay medium to measure Lactococcus lactis enzyme activities while mimicking intracellular conditions. *Applied and Environmental Microbiology*, *78*(1), 134–143. https://doi.org/10.1128/AEM.05276-11

Machado, D., Andrejev, S., Tramontano, M., & Patil, K. R. (2018). Fast automated reconstruction of genome-scale metabolic models for microbial species and communities. *Nucleic Acids Research*, *46*(15), 7542–7553. https://doi.org/10.1093/nar/gky537

Oliveira, A. P., Nielsen, J., & Förster, J. (2005). Modeling Lactococcus lactis using a genome-scale flux model. *BMC Microbiology*, *5*(1), 39. https://doi.org/10.1186/1471-2180-5-39
