# Supplementary material for: High-throughput generation and comparison of genome-scale metabolic models reveal strain-specific metabolic diversity in 439 Lactococcus strains
Source: mSystems. 2026 Mar 30;11(4):e01517-25. doi: 10.1128/msystems.01517-25 (PMC13098203; doi:10.1128/msystems.01517-25)
Supplement: Text S2 — Detailed description of additional curations for generated 439 Lactococcus models. [file msystems.01517-25-s0002.docx]

# Text S2: Detailed description of additional curations for generated 439 *Lactococcus* models

## 1) Additional curation

Additional curation of the GEMs was needed to improve the model simulation output. Instead of curating each strain specific GEM individually, the reactions from all strain specific GEMs were compiled into a single model, often referred to as a “panmodel”, which was subsequently used to identify reactions or pathways that needed curation. This was combined with the simulation results for each of the strain specific models.

The following limitations were identified that needed curation:

1. Several of the GEMs were found to contain energy generating loops, resulting in unrealistically high biomass yields.
   1. Energy generating loops created by transport reactions: Several energy generating loops were found to be caused by models containing multiple transport reactions for the same metabolite, for which some transported protons (symport or antiport) while others did not (see supplementary figure B1a for an example). This results in a proton gradient over the membrane which is used by the ATP-synthase for ATP generation. Each identified loop was evaluated manually and resolved depending on the situation by the solutions listed below.

**Curation:**

- - 1. If only one of the transport reactions was connected to genes, the reaction that was not connected to genes was selected to be removed from models containing this reaction.
    2. Duplicate reactions with differences in reaction directionality were merged, for example one export and one uptake reaction both using proton symport were merged into one bidirectional reaction. These reactions were mostly associated with the same genes, however if needed, genes associated with these reactions were both connected to the merged reaction.
    3. The reaction bounds of the remaining reaction were changed to allow the reaction to facilitate both uptake and export.
    4. For strain specific GEMs that only contained one of the duplicate reactions, this reaction was also adjusted to match the selected and/or updated transport reaction.
  1. Energy generating loop due to the presence of 2 reversible ATP-synthase reactions with different proton stoichiometry. This can be used to generate a proton gradient over the membrane resulting in ATP generation (see supplementary figure B1b) for example).
     **Curation:**
     1. It was assumed that the proton stoichiometry for ATP-synthase was the same for all *Lactococcus* strains used in this study. To that end one specific ATP-synthase reaction was selected which was then set as the ATP-synthase reaction in all strain specific GEMs. The selected ATP synthase was the ATP synthase utilized in the MG1363 model (Flahaut et al., 2013).
     2. The other ATP synthase reaction was removed from the strain specific GEMs. Connected genes to other ATP synthase reactions were added to the selected ATP synthase reaction.

1. The GEMs were unable to produce several common fermentation products such as acetate and formate. This was caused by that the transport reactions for these products were constrained to allow only uptake of these products and no export (see figure B1c) for example).
   **Curation:**
   1. The reaction bounds for transport reactions for common fermentation products were manually evaluated and adjusted if needed to allow for product export.
   2. Proton symport, proton antiport and (facilitated) diffusion reactions were assumed to be bidirectional unless otherwise specified.
   3. Energy requiring transport mechanisms such as the phosphotransferase system (PTS) and the ATP depended ABC-transporter systems were assumed to be one-directional.


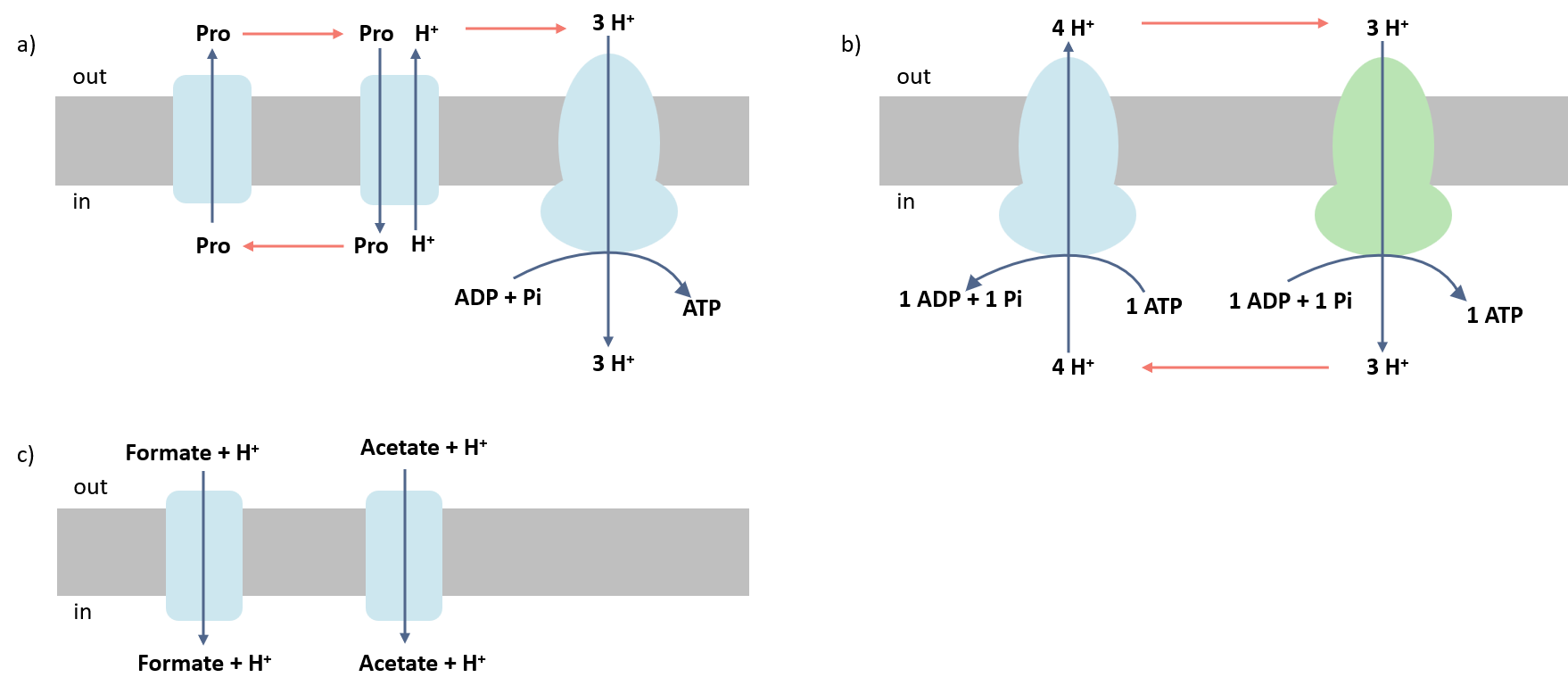


Figure B1: a) Example of an energy generating loop. Proline is taken up by proton antiport, while it can be exported via another reaction without translocating a proton. This allows the network to create a proton gradient over the membrane which can be used by the ATP synthase to generate ATP. b) Example of energy generating loop caused by two different ATP-synthase reactions with different proton stoichiometry. The left ATP synthase reaction has a proton stoichiometry of 4, whilst the right has a proton stoichiometry of 3. If the blue ATP synthase reaction works reversed, 4 protons are translocated over the membrane by using one ATP. The green ATP synthase reaction then uses this proton gradient to create ATP using only 3 protons, so it can make 1,33 ATP per one ATP invested in the blue ATP synthase.
c) Example of reactions that needed a directionality change. Under shown conditions Formate and acetate could only be taken up and not exported, so they could not be produced as fermentation products.
