# Supplementary material for: High-throughput generation and comparison of genome-scale metabolic models reveal strain-specific metabolic diversity in 439 Lactococcus strains
Source: mSystems. 2026 Mar 30;11(4):e01517-25. doi: 10.1128/msystems.01517-25 (PMC13098203; doi:10.1128/msystems.01517-25)
Supplement: Supplemental Figures — Figures S1-S12. [file msystems.01517-25-s0003.docx]

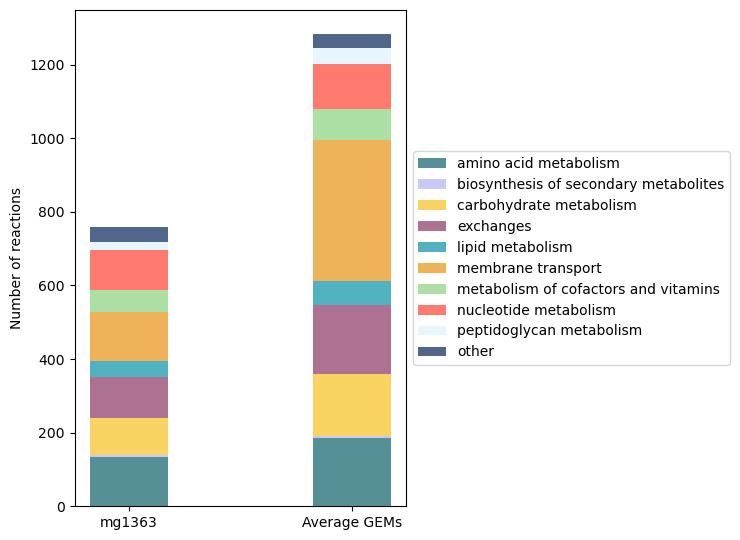


Figure S1: Barplot of the distribution of reactions over different pathway categories for the reactions in the MG1363 model compared to the average of the GEMs created in this study.


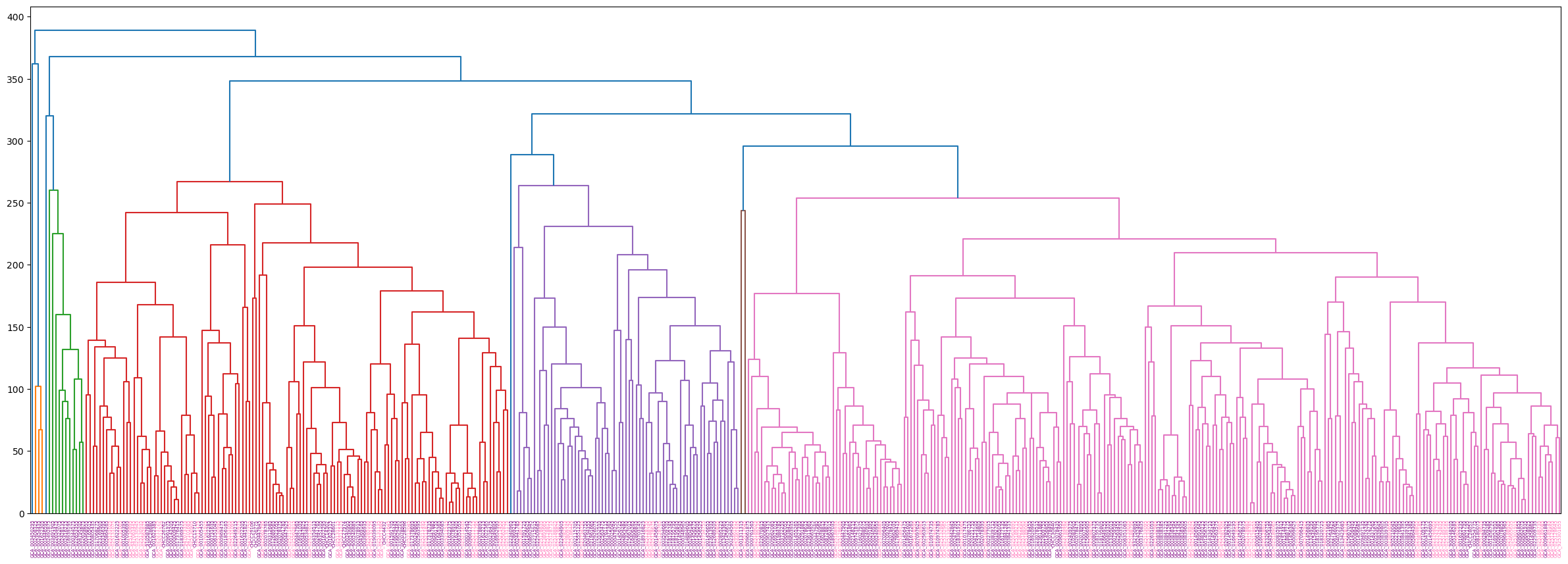


Figure S2: Clustering of the strain specific GEMs based on reaction content. Models generated for the first set of genomes are indicated in purple. The 114 additional models generated for the second set of genomes added to NCBI are indicated in pink.


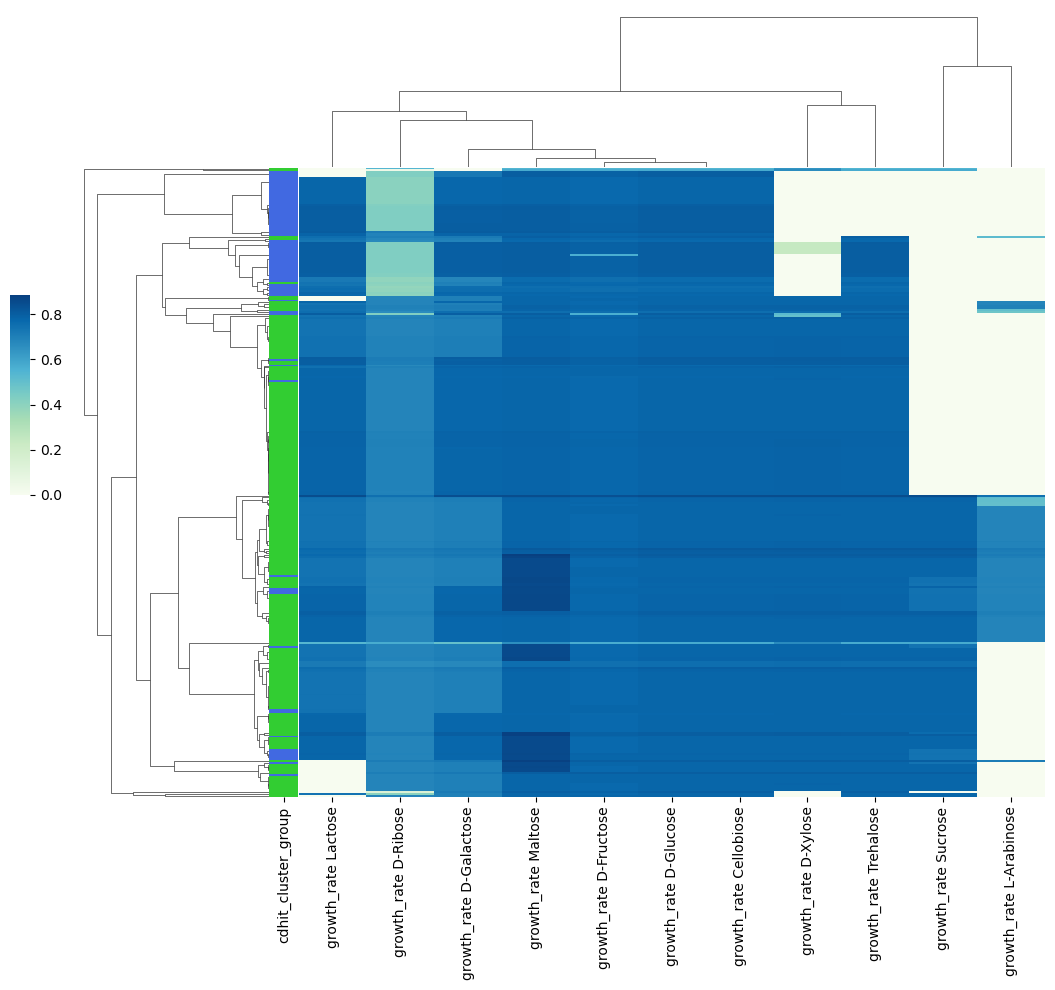


Figure S3: Models clustered based on predicted biomass growth rate h^-1^ determined for c-mol equivalents to 20 mmol g^-1^ h^-1^ glucose for different substrates. The color shows model species, L. lactis is indicated in green, L. cremoris is indicated in blue.


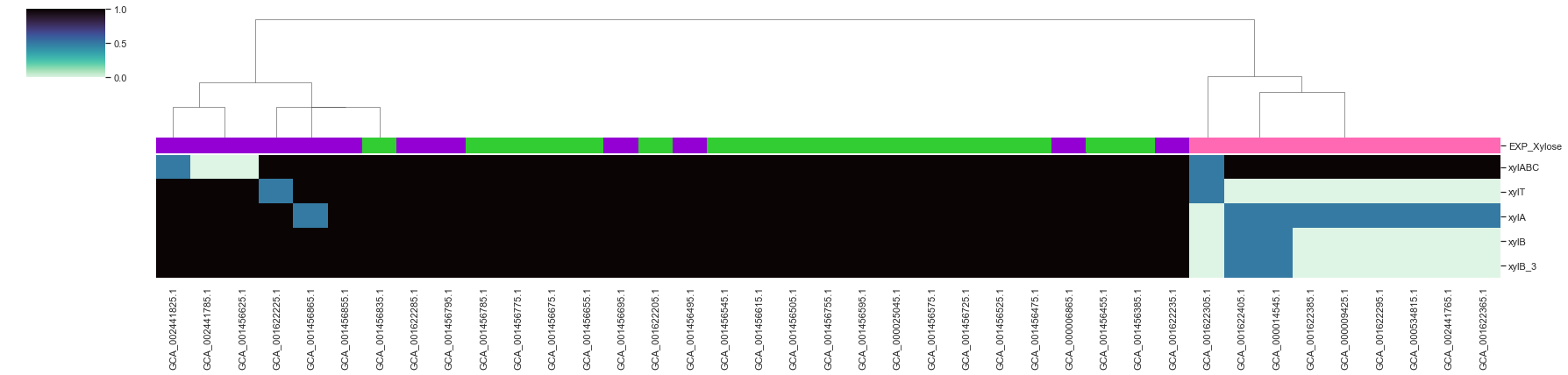


Figure S4 Gene presence absence of xylose uptake and utilization genes for the 36 strains from the phenotypic dataset by Bayjanov et al. Black indicates gene presence, blue indicates presence of a truncated homologue that is potentially a pseudogene. The color row indicates growth on xylose, predicted by the GEM and based on experimental values from Bayjanov et al., green indicates that strains are xylose positive both experimentally and predicted by the GEM, pink indicates xylose negative both experimentally and based on GEM predictions, purple indicates xylose negative based on experimental data, but xylose positive based on GEM predictions. xylABC: ATP dependent xylose transporter, xylT: xylose transferase, xylA: xylose isomerase, xylB: xylulokinase, xylB_3: alternative xylulokinase.


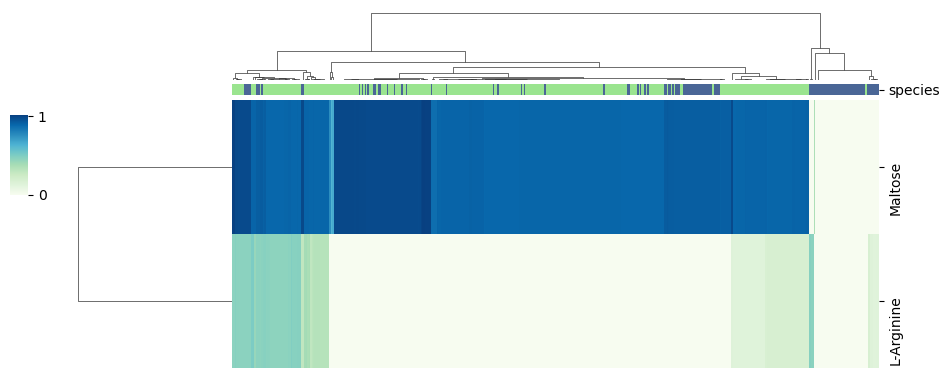


Figure S5: Models clustered based on predicted biomass growth rate h^-1^ determined for c-mol equivalents to 20 mmol g^-1^ h^-1^ glucose for Maltose and L-Arginine, when constraining the uptake of maltose in the ATP-dependent maltose transporter. The color shows model species, L. lactis is indicated in green, L. cremoris is indicated in blue.


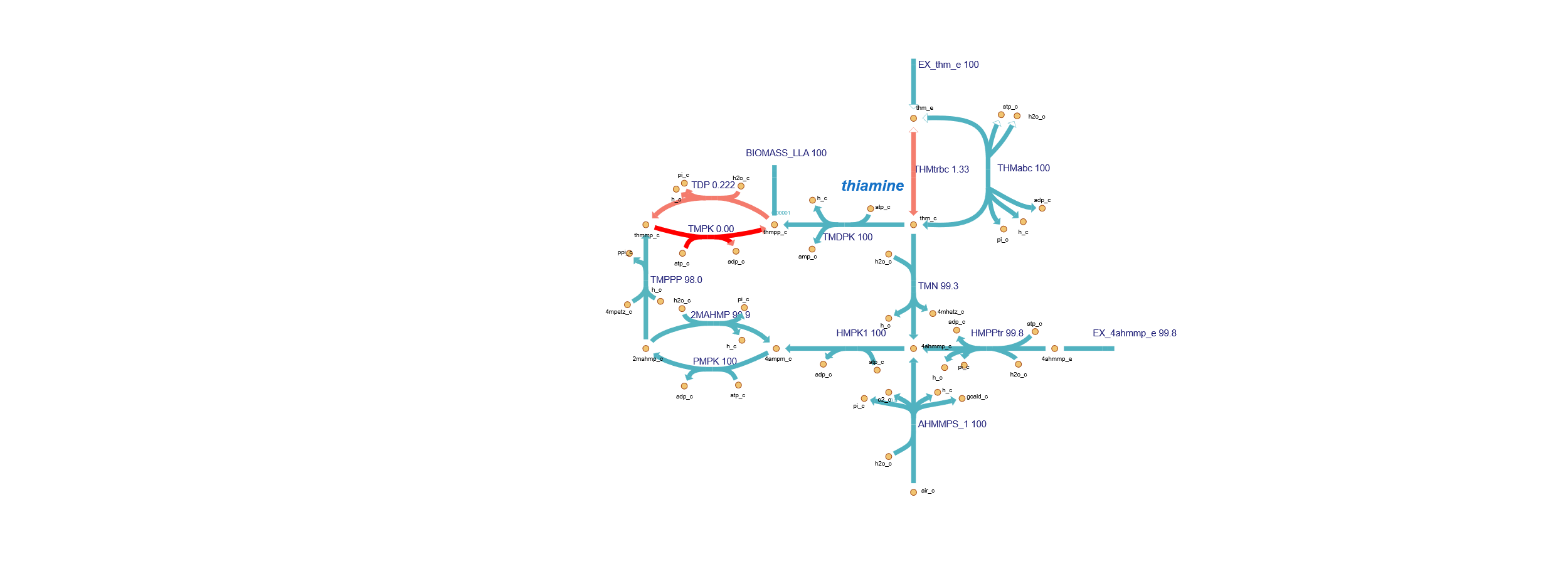


Figure S6: metabolic pathway map of Thiamine biosynthesis and metabolism. The values shown with the reaction ids represent the percentage of the 451 GEMs containing this reaction.


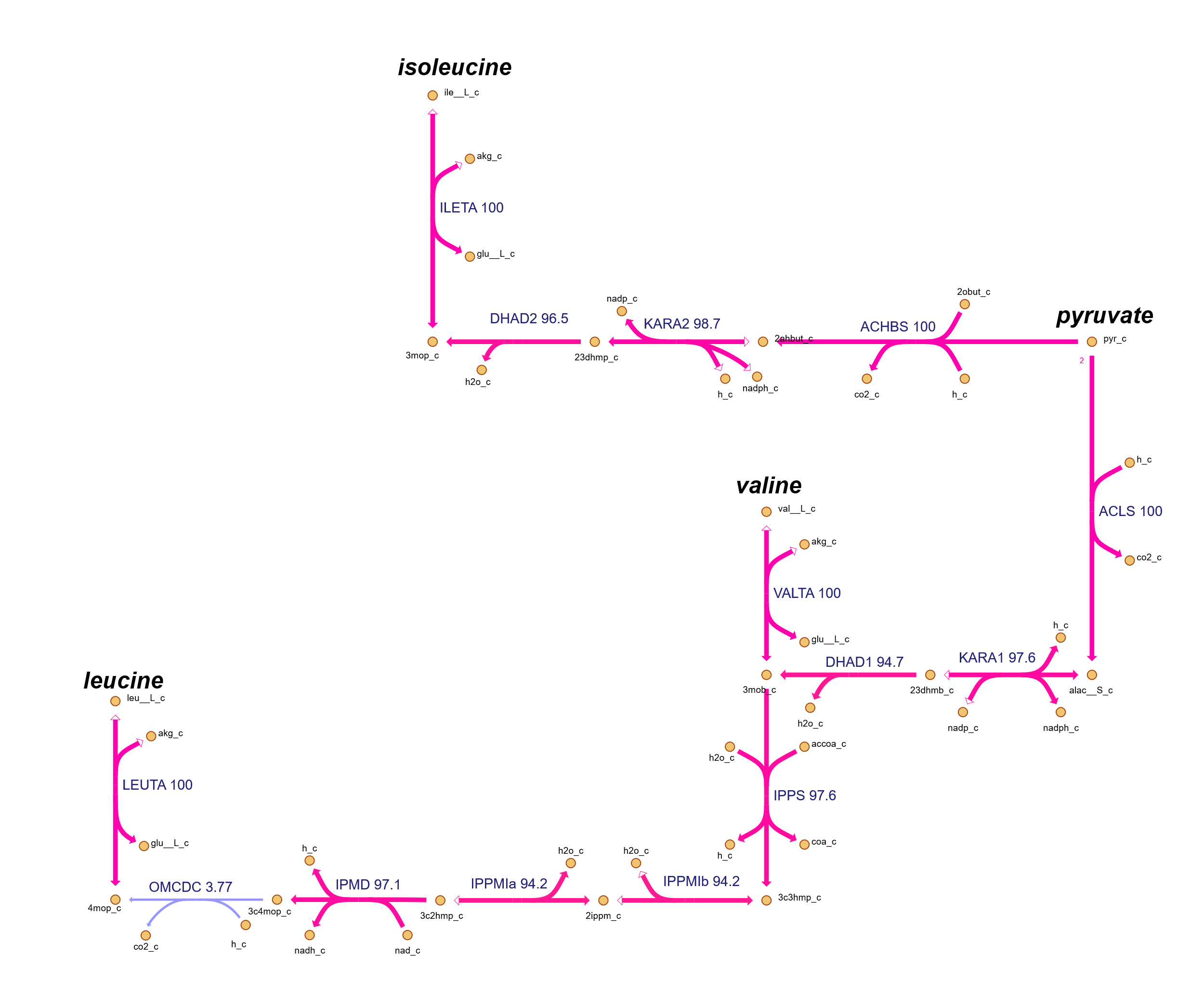


Figure S7: Biosynthesis pathways for the branched-chain amino acids, valine leucine and isoleucine. The value on the reaction indicates the percentage of GEMs that contain this reaction.


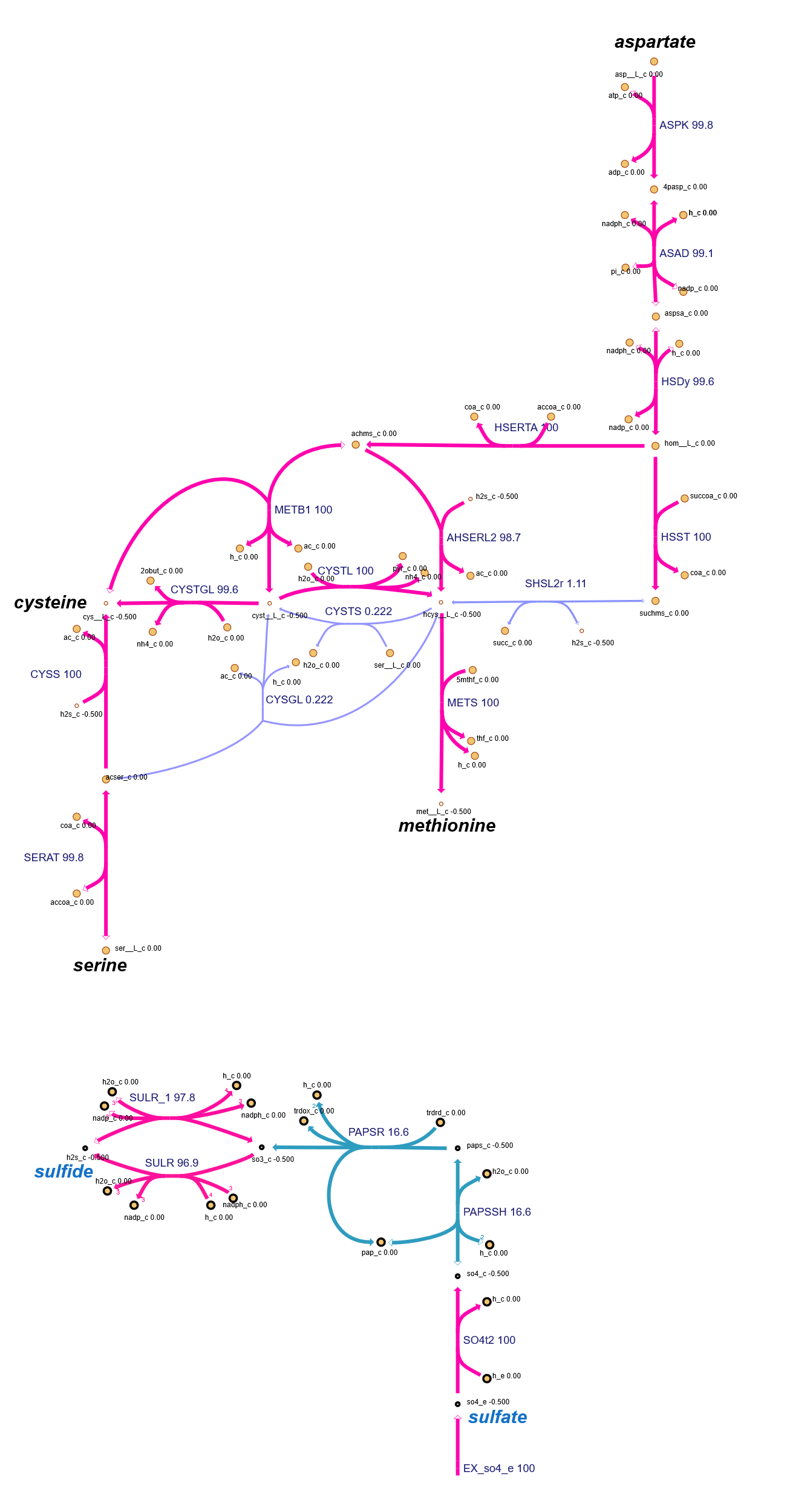


Figure S8: Cysteine and Methionine pathways. The value on the reaction indicates the percentage of models that contain this reaction.


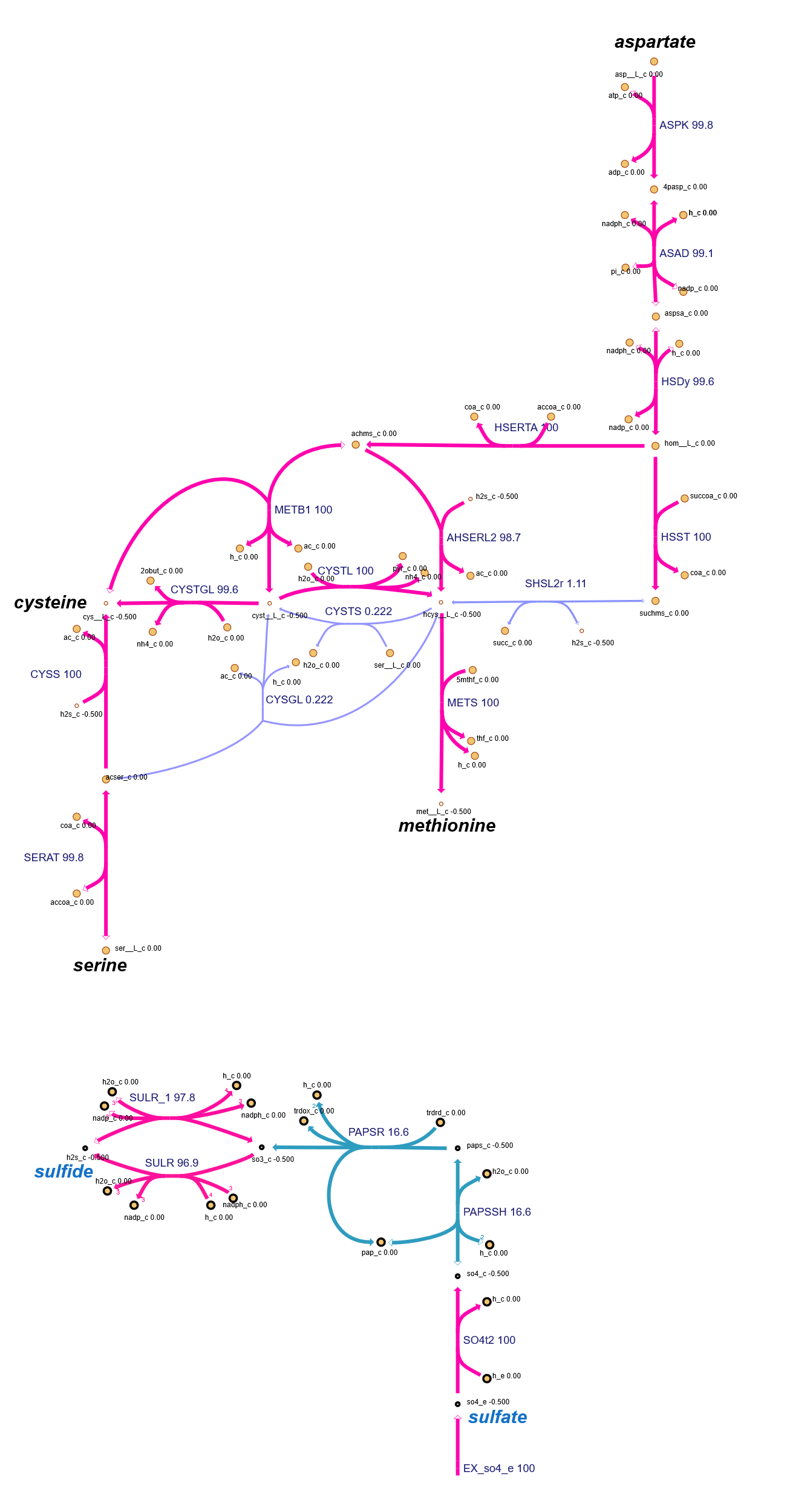


Figure S9: Reactions for converting sulfate to sulfide, which is then used in the cysteine and methionine biosynthesis pathways


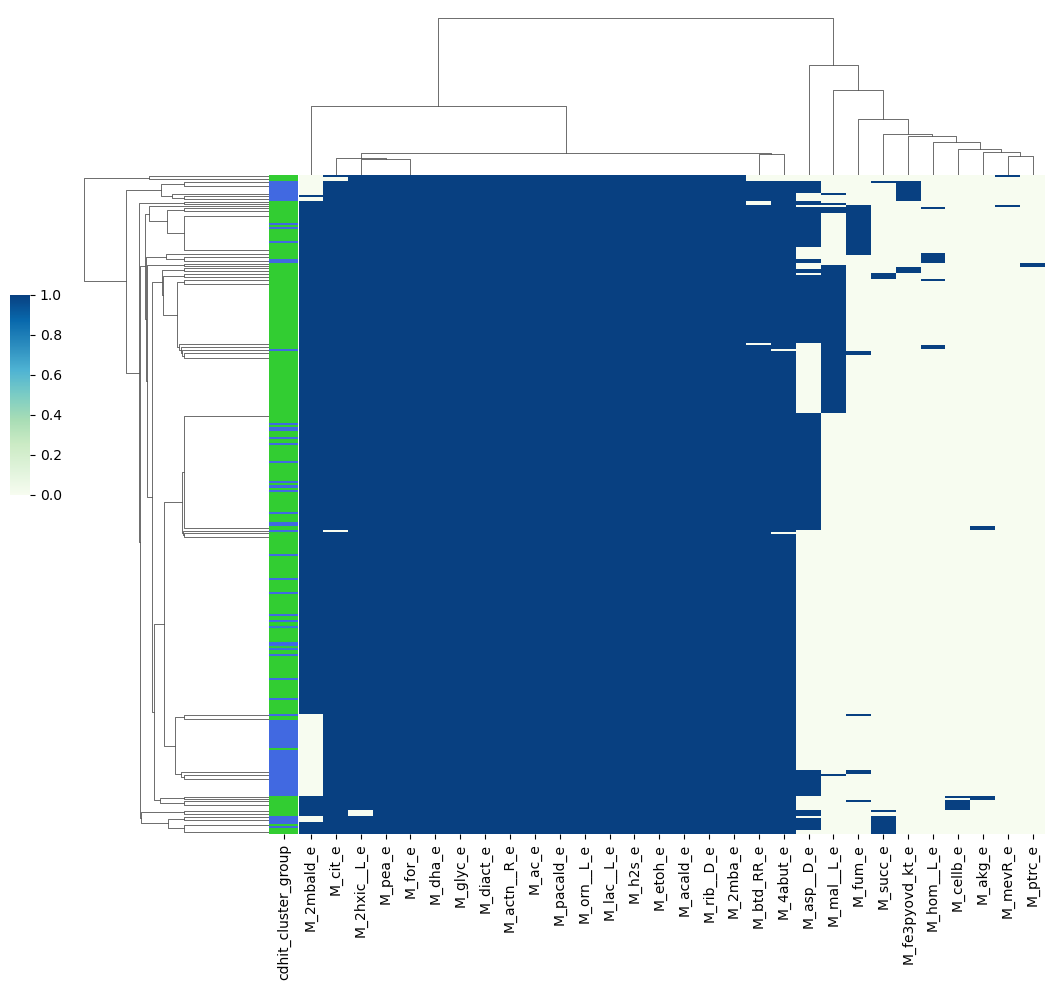


Figure S10: Smetana predictions product donor, blue color indicates each of the strains being predicted as a donor of the metabolites listed at the x-axis.


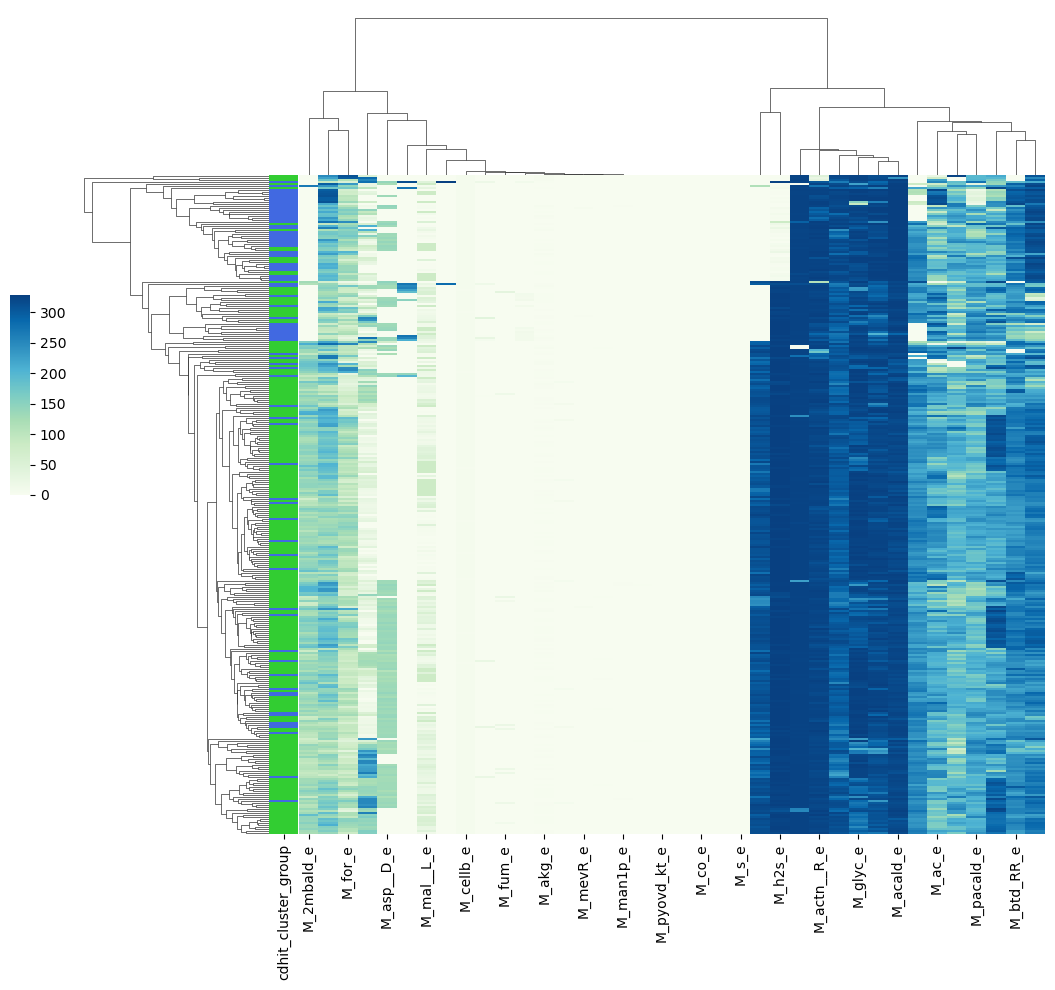


Figure S11: Smetana predictions metabolite receivers, blue color indicates each of the strains being predicted as a receiver of the metabolites listed at the x-axis.


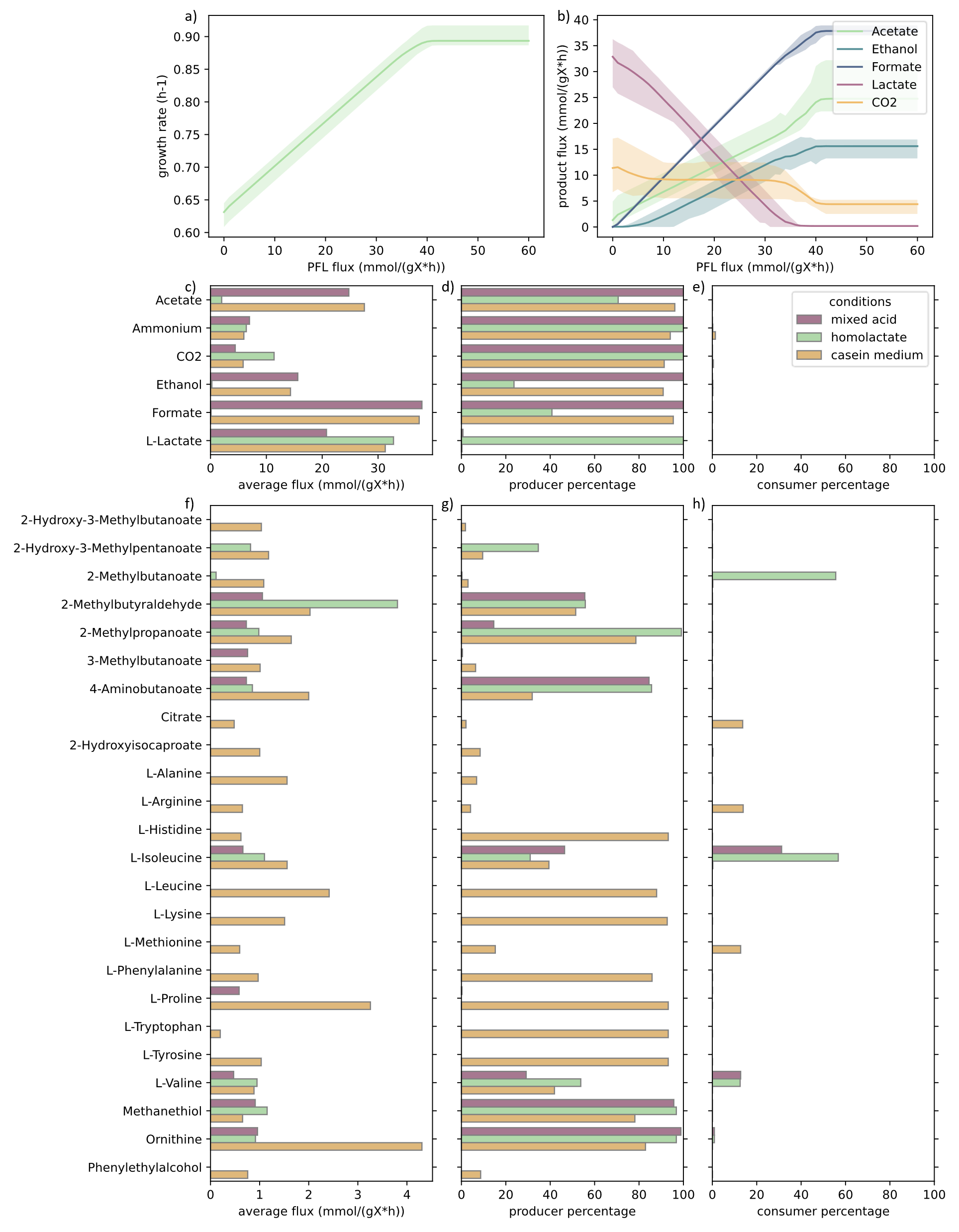


Figure S12 a) The distribution of biomass yield predicted for L. lactis (green) and L. cremoris (blue) models for mixed-acid fermentative growth. b) the distribution of biomass yield predicted for L. lactis (green) and L. cremoris (blue) models for homolactate fermentative growth. The biomass yield value of the template L. cremoris model (Flahaut et al. 2013) for mixed acid and homolactate fermentation conditions is shown by the black marker.
c and d) Effect of constraining the PFL flux on biomass growth rate (c) and product formation (d). Average flux for all models is shown as a solid line, and the corresponding shaded ranges represent the fluxes between 5 and 95% percentile of all predictions. b) shows the biomass specific production rates for Acetate, Ethanol, Formate, Lactate and CO2 (mmol g^-1^ h^-1^).
e, f, g and h) overview of the fermentation products predicted by the model simulations for mixed acid fermentation on CDM (purple), homolactate fermentation on CDM (green) and mixed-acid fermentation on casein medium (yellow).

c) Mean product fluxes producing strains for the main fermentation products under set conditions. d) percentage of models producing the product for main fermentation products. e) percentage of models that consumes this product when present in the medium, indicating potential for cross-feeding . f) Mean product fluxes for producing strains for fermentation byproducts under set conditions described above. g) percentage of models producing the product for fermentation byproducts. h) percentage of strains specific GEMs that consume the fermentation product when present in the medium, indicating potential for cross-feeding.
