# Supplementary material for: High-throughput generation and comparison of genome-scale metabolic models reveal strain-specific metabolic diversity in 439 Lactococcus strains
Source: mSystems. 2026 Mar 30;11(4):e01517-25. doi: 10.1128/msystems.01517-25 (PMC13098203; doi:10.1128/msystems.01517-25)
Supplement: Supplemental Table Legends — Legends for Tables S1-S11. [file msystems.01517-25-s0004.docx]

**Table S1:** List of the L. lactis and L. cremoris genomes used in this study. The following information is provided: GCA id, species, cdhit cluster group, subspecies, species indicator, strain information, source metadata, assembly accession, FTO path, and ASM name.

**Table S2:** Overview of the curated metabolites in the universal bacterial model, including id, name, formula, charge and annotations. In each case, it’s highlighted if they were added, changed or removed.

**Table S3:** Overview of the curated reactions in the universal bacterial model, including id, name, formula, balance status and annotations. In each case, it’s highlighted if they were added, changed, removed or merged with duplicate.

**Table S4:** Overview of the modifications to metabolites to the MG1363 model, including id, name, formula, charge and annotations. In each case, it’s highlighted if they were added, changed, removed or merged with duplicate.

**Table S5:** Overview of the modifications to the reactions of the MG1363 model, including id, name, formula, balance status and annotations. In each case, it’s highlighted if they were added, changed, removed or merged with duplicate.

**Table S6:** Media composition file, showing the compound and name for each of the 3 media types used.

**Table S7:** Overview of the curations to the generated models, including the corresponding reaction and the type of modification performed.

**Table S8:** Simulation results of predicted auxotrophies for different medium components. The values represent the percentage of biomass yield achieved on medium without each medium component compared to complete medium composition. Therefore, a value of 100 signifies the strain is not auxotroph to the metabolite (grows in the same way without the metabolite), and a value of 0 signifies the strain is fully auxotroph.

**Table S9:** Overview of the representation of reactions linked to maltose uptake and utilization in the 439 GEMs for L. lactis and L. cremoris strains generated in this study. The table shows the number of models that contain the reaction and the number of models in which the reaction has genes connected to the reaction.

**Table S10:** The essentiality of all medium compounds was evaluated for each of the models, table showing lowest growth rate observed (min growth rate) in the models, the highest growth rate observed in the models (max growth rate) and the mean growth rate observed in the models. If the maximum growth rate was equal to 0, the component was considered essential in all strains, if both min. and max. growth rates were positive non-zero values, the component was considered not essential for all strains. However, if the growth rates with leaving out the component were only equal to 0 for some strains, the essentiality of the component varied between different strains (indicated as mixed).

**Table S11:** reactions involved in cysteine and methionine biosynthesis, indicating the percentage of models that have genes connected to these reactions and the percentage of strain specific models containing each of these reactions.
